# Combining TMS and tACS for Closed-Loop Phase-Dependent Modulation of Corticospinal Excitability: A Feasibility Study

**DOI:** 10.3389/fncel.2016.00143

**Published:** 2016-05-25

**Authors:** Valerio Raco, Robert Bauer, Srikandarajah Tharsan, Alireza Gharabaghi

**Affiliations:** Division of Functional and Restorative Neurosurgery, and Centre for Integrative Neuroscience, Eberhard Karls University TübingenBaden-Württemberg, Germany

**Keywords:** brain state-dependent, phase-dependent, adaptive, targeted modulation, beta oscillations

## Abstract

**Background**: The corticospinal excitability indexed by motor evoked potentials (MEPs) following transcranial magnetic stimulation (TMS) of the sensorimotor cortex is characterized by large variability. The instantaneous phase of cortical oscillations at the time of the stimulation has been suggested as a possible source of this variability. To explore this hypothesis, a specific phase needs to be targeted by TMS pulses with high temporal precision.

**Objective**: The aim of this feasibility study was to introduce a methodology capable of exploring the effects of phase-dependent stimulation by the concurrent application of alternating current stimulation (tACS) and TMS.

**Method**: We applied online calibration and closed-loop TMS to target four specific phases (0°, 90°, 180° and 270°) of simultaneous 20 Hz tACS over the primary motor cortex (M1) of seven healthy subjects.

**Result**: The integrated stimulation system was capable of hitting the target phase with high precision (SD ± 2.05 ms, i.e., ± 14.45°) inducing phase-dependent MEP modulation with a phase lag (CI95% = −40.37° to −99.61°) which was stable across subjects (*p* = 0.001).

**Conclusion**: The combination of different neuromodulation techniques facilitates highly specific brain state-dependent stimulation, and may constitute a valuable tool for exploring the physiological and therapeutic effect of phase-dependent stimulation, e.g., in the context of neurorehabilitation.

## Introduction

Transcranial magnetic stimulation (TMS) is capable of probing corticospinal excitability, modulating brain activity and disrupting pathological patterns (Hallett and Chokroverty, 2005; Siebner and Ziemann, 2007; Chen et al., 2008). However, there is a physiological trial-to-trial variability in motor-evoked potential (MEP) amplitude following identical TMS pulses most likely related to the brain state at the time of stimulation (Kiers et al., 1993; Thickbroom et al., 1999; Darling et al., 2006). A solid understanding of the interplay of stimulation effects with the underlying cortical physiology is crucial to the reliable implementation of this technology in a therapeutic setting. TMS has therefore been combined with electroencephalographic (EEG) recordings to explore this interaction. There is increasing evidence that the prestimulus cortical power (mainly in the alpha and beta range) has a significant influence on the MEP (Zarkowski et al., 2006; Lepage et al., 2008; Sauseng et al., 2009; Mäki and Ilmoniemi, 2010; Feurra et al., 2013; Takemi et al., 2013; Gharabaghi et al., 2014; Kraus et al., 2016a,b). In addition, recent studies have applied different methodologies to explore the influence of the prestimulus phase of cortical rhythms on the MEP (Ferreri et al., 2011; Keil et al., 2013; Schulz et al., 2014; Berger et al., 2014; Kundu et al., 2014). The estimation of phase-dependency is challenged by the necessity to acquire evenly distributed TMS pulses across the phase spectrum to reduce any bias due to unequal distribution of the sampled phases. Many studies therefore applied a time jitter between stimulation pulses (Ferreri et al., 2011; Keil et al., 2013; Schulz et al., 2014; Berger et al., 2014; Kundu et al., 2014) instead of fixed time-intervals (van Elswijk et al., 2010). However, to evaluate this data, different analysis methods such as Fourier (Mäki and Ilmoniemi, 2010; van Elswijk et al., 2010), Hilbert (Keil et al., 2013) or Wavelet transformation (Berger et al., 2014) were applied, making it difficult to draw direct comparisons between the different results.

One alternative to a *post hoc* analysis of the interaction of randomly applied stimuli and the corresponding brain state is to apply the pulses in a more controlled way, e.g., by triggering them on the basis of online detection of the current phase. By applying adaptive thresholding of the brain signal in the time-domain, for example, stimuli were directed towards the peak and trough of low frequency oscillations (0.16 and 2 Hz) during sleep (Bergmann et al., 2012). Zrenner et al. (2015a,b) recently proposed the use of dedicated real-time recording and analysis hardware for phase-locked stimulation in the alpha-range on the basis of forward projection of a sliding window Fourier-transformation approach. Since any triggering is subject to an inherent time lag and is based on noisy measurements in a dynamical system, phase-dependent stimulation faces several obstacles. On the basis of features of the measured data, a predictive model of the underlying brain activity has first to be developed (predictability problem). Secondly, the speed of the technical system, mainly determined by the delay of signal analysis and triggering, must be faster than the dynamics of the target feature (real-time problem). Finally, the timing of the whole system must be precise enough to successfully target the desired features, i.e., phase jitter must be low (precision problem). Phase-dependent stimulation is also affected by the issue of a methodological flexibility (albeit less than *post hoc* approaches) during estimation of the phase spectrum. While all transformation methods estimating the instantaneous phase may, in theory, provide equal results (Bruns, 2004), their flexibility with regard to the exact implementation may cause inferential problems (Gelman and Loken, 2014).

To overcome the above-mentioned problems, we propose the combination of two non-invasive brain stimulation methods to study the dependency of stimulation effects on the phase of cortical oscillations. Specifically, we used transcranial alternating current stimulation (tACS) to modulate the spontaneous oscillatory activity, thus addressing the predictability and real-time problem. Moreover, to deliver TMS at the desired phase of the tACS, calibration of the systematic time-lag was applied, thereby addressing the precision problem. The basic concept of combining tACS with TMS has already been applied, e.g., to assess pre-post changes in cortical excitability following repetitive stimuli (Goldsworthy et al., 2016). It has also been used at a very low tACS frequency (0.8 Hz) with a positive current offset (Bergmann et al., 2009). Here, we extend this line of research by implementing synchronous recording of the tACS signal and the TMS artifact to assess and calibrate the temporal precision of the applied single pulses in relation to oscillations at a higher frequency than has ever been studied before, i.e., in the beta band (20 Hz). As well as testing its methodological feasibility, we also aimed to exploit the temporal precision of this approach by studying phase specific modulation of corticospinal excitability.

## Materials and Methods

### Subjects

Having given written informed consent, seven healthy subjects (mean age: 22 years, STD: 3 years; 5 males; all right handed) took part in this methodological feasibility study which is part of a larger ongoing study. None of the subjects had any history of neurological diseases or medication. The study protocol was approved by the local Ethical Committee of the medical faculty of the University of Tübingen and was carried out in accordance with the principles of the Declaration of Helsinki.

### Preparation

**Figure 1:**
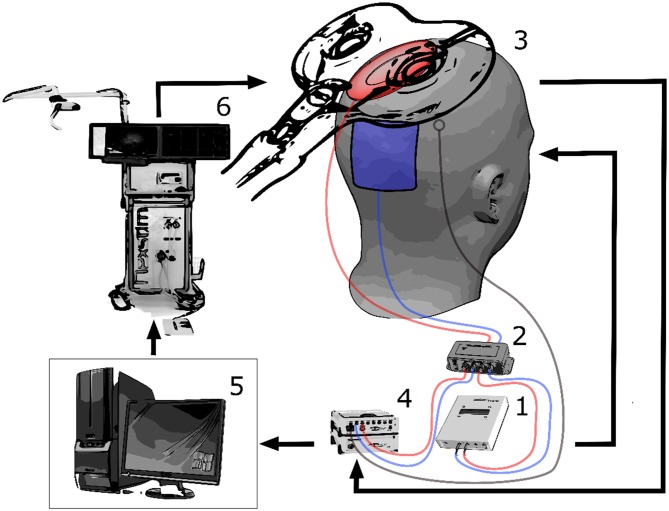
**The experimental setup is shown.** The alternating current stimulation (tACS) stimulator (1) is connected to a current divider (2) that re-routes a part of the tACS signal directed to the subject (3) back to the electroencephalographic (EEG) amplifier (4) for recording. The recording computer (5) also triggers the transcranial magnetic stimulation (TMS) system (6). The stimulation artifact is recorded via an EEG electrode positioned on the subject’s head. By converging the two stimulation artifacts to the controlling phase-consistency (PC), a precise synchronization of the whole system can be carried out after a test pulse. Thereafter, TMS pulses can be applied at specific phases of the tACS waveform.

Bipolar electromyography (EMG) recording of the first dorsal interosseous (FDI) muscle of the right hand was performed in belly-tendon montage with a sampling rate of 5 kHz (BrainAmp ExG, Brain Products, Munich, Germany). We determined the location of the FDI hotspot in the primary motor cortex (M1) as the spot that elicits the highest MEP with the lowest TMS intensity. TMS was delivered by an integrated neuro-navigated system (Nexstim, Helsinki, Finland) with a figure-8-shaped coil that induced a posterior-anterior current flow. Once the hotspot had been determined, a rubber ring electrode (internal diameter 2.5 cm, external diameter 5 cm) was positioned over the hotspot and a second rectangular electrode (5 × 6 cm) was positioned over Pz. Both electrodes were attached to a DC/AC stimulator (NeuroConn, Ilmenau, Germany) and electrolyte gel was used to keep the impedance below 10 KΩ. The electrodes were kept in place by a tight EEG cap that covered the scalp. In addition, a fraction of the tACS signal current was routed via current division (1 MΩ vs. 1 kΩ) and subsequently recorded using a bipolar amplifier with 5 KHz sampling rate. Since the amplifier’s input resistance was 10 GΩ, the current lost to recording was negligible. Furthermore, we added two passive Ag/Ag-Cl-electrodes next to the hotspot position, i.e., directly under the TMS-coil, to detect any artifacts. Having positioned the stimulation electrodes, we used the neuro-navigated TMS system to keep coil position and orientation constant over the determined hotspot during the subsequent measurement and intervention. We assessed the resting motor threshold (RMT) of the FDI, using a staircase procedure to detect the TMS intensity inducing MEPs above 50 μV in 50% of the pulses. We calculated six stimulation intensities (SI) at 90%, 100%, 110%, 120%, 130% and 140% relative to the RMT for each subject. The setup is shown in Figure 1.

### Technical Procedure

The intervention was performed in six runs, in each of which TMS was applied at a different SI. The order of the SI of each run was randomized across subjects. In the present methodological feasibility study, we report the findings during the SI of 110% only. Each run lasted around 3 min, with a 1-min break between runs. During each run, 200 s of tACS (20 Hz, 1 mA, 1 s ramp-up, 1 s ramp-down) were delivered to the subject, limiting the total stimulation duration of the study to 20 min (Nitsche and Paulus, 2007). In earlier research, we observed that 20 Hz tACS are liable to induce phosphene sensations (Raco et al., 2014). However, none of the subjects in this study reported neurosensory effects.

**Figure 2:**
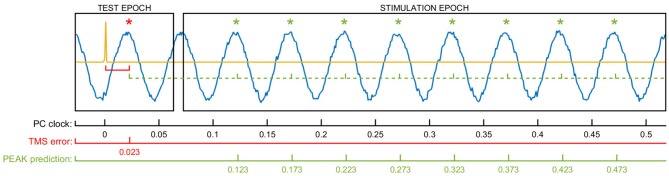
**The figure shows exemplary data used for the phase-specific stimulation algorithm and the respective variables involved in the calculations.** The yellow signal represents the TMS artifact of the test pulse delivered randomly at the beginning of the epoch. The sinus line shows the recorded raw tACS waveform. The delay between the TMS pulse and the first target phase in the data (TMS error) is used to calculate the future time windows to trigger the TMS at the specific tACS phase. In the example shown here, the 23 ms TMS error is added to a multiple of the stimulation cycle time (50 ms) to detect the tACS peaks (PEAK prediction). By using this method, the delays connected to streaming of the data and the triggering of both TMS and tACS are implicitly considered in the calculation and don’t need to be addressed separately.

At the beginning of each run, we used a series of TMS test pulses to synchronize tACS phase and TMS stimulation timing. Following calibration (see below), TMS pulses were triggered at the run-specific intensity every 5 s (±500 ms predefined jitter) while targeting one of four specific tACS phases: peak, falling flank, trough, and rising flank (i.e., 0°, 90°, 180° and 270°) in random order. Each of these four phases was targeted at random 10 times during each run, resulting in a total of 40 stimulation pulses per run. To achieve the necessary precision, we synchronized the two stimulators using a closed-loop automatic calibration lasting for approximately 1 s at the beginning of each run. This procedure is specified in the code below. For this calculation, a random TMS pulse was briefly triggered at the onset of the tACS while the phase that was hit by this first TMS test pulse was analyzed. This enabled us to estimate the time/phase-lag of the stimulation system following the pseudo-code which illustrates the applied algorithm in detail, Moreover, exemplary signal fed to the algorithm is shown in Figure 2.

### Pseudo-Code for Hardware Synchronization

%% TEST PULSE AND HARDWARE SYNCHRONIZATION

Start tACS

Start recording

Initialize clock

Deliver TMS test pulse

Determine tACS phase of TMS

for *n* = 1 : number_of_trials

Wait for defined inter-trial-interval (plus jitter)

Determine current tACS phase based on clock

Select target phase from a (permuted) set of phases

Calculate shortest waiting time necessary to hit target phase with TMS

Wait for the waiting time

Trigger_TMS_pulse

end

### Preprocessing and Analysis

The recorded EMG data was divided in epochs, with a time range of ±500 ms centered on the TMS artifact. The data was visually inspected, and trials contaminated by artifacts, and thus preventing the detection of MEPs, were removed (minimum number of trials removed per subject: 1, mean: 2.1, maximum: 4, total: 15, percentage of all trials: 1.5%). The peak-to-peak amplitude of the MEPs was measured as the range of the EMG trace from 10 to 50 ms following the TMS pulse. Within each subject, MEP amplitudes were normalized relative to the MEP amplitude at the 95th percentile of all measured MEPs. We averaged the MEPs over windows, i.e., for the first three and last three trains.

Please note that, although the stimuli were applied in random order, their distribution over the tACS waveform was even. Since they translate to a period length N of 4, we were subsequently able to apply discrete Fourier transformation to the MEP values to estimate magnitude and phase-lag of the interaction between tACS phase and TMS effect. The complex values could also be used to estimate the coherence of the phase-lag across subjects in a manner similar to that for inter-trial coherence (ITC). We began by transforming the phase of every subject to a vector on the unit circle according to the formula (1):

(1) $$\hat{x}\;=\;e^{\left(1i*\theta (x)\right)}$$

where $\hat{x}$ represents a unit-length complex value, *e* is the Euler’s number and θ($\vec{x}$) represents the angle of the original complex value. Since we wished to test the phase-consistency (PC) across subjects, we took the absolute value of the mean of $\hat{x}$ across subjects using the following formula (2), where *N* is the number of subjects:

(2) $$PC\;=\;\left|\frac{1}{N}\sum_{i\;=\;1}^{N}\hat{x}\left(n\right)\right|$$

PC is bound to the range between 0 (no coherence) and 1 (full coherence) and can be understood geometrically as the length of the mean vector. This length represents the stability of the phase-dependent MEP modulation across the subjects. To assess statistical significance, we permuted 1000 times the four MEP values for each subject and repeated the analysis. We considered the MEPs to be significantly modulated by the tACS phase when the actually measured phase consistency exceeded the 95th percentile of the distribution with permutation.

### System Precision

**Figure 3:**
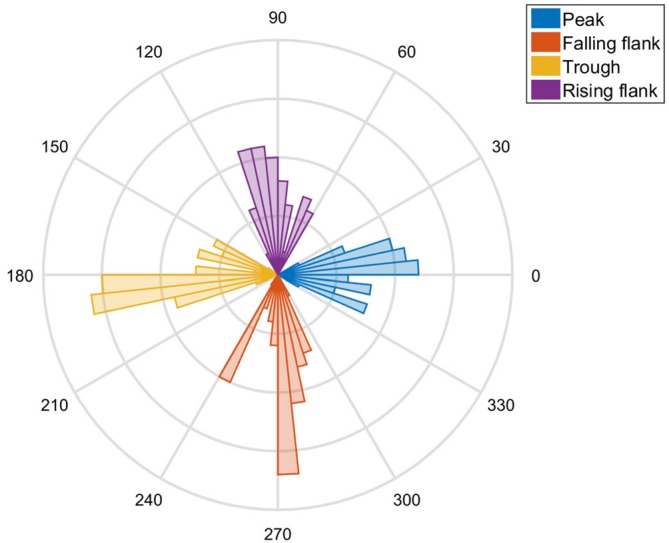
**A polar plot of the tACS phases hit by the TMS in all subjects is shown.** Clear peaks at 0°, 90°, 180° and 270° are visible as evidence of the precision of the method.

To assess the precision of the system, we concatenated the trials of the seven subjects. We assessed the phase of the actual stimulation on the basis of a Fourier transformation of the 500 ms prior to the TMS pulse. The distribution is illustrated by a histogram (Figure 3). We then shifted the actual phase measured by the targeted phase of that trial (i.e., 0°, 90°, 180° and 270°) and used the CircStat toolbox (Berens, 2009) to assess the confidence intervals.

## Results

### Phase and Temporal Precision

Visual inspection of the distribution revealed that the actual phase angle did indeed exhibit a distribution centered on the anticipated angle (Figure 3). The targeted phase was well within the confidence intervals of the distribution of the stimulated phases. The data of the seven subjects suggests that the phase lag was not significantly different from zero, indicating that there was no systematic bias (*p* = 0.65). The combined stimulation system was capable of hitting the target phase with high temporal precision (SD ± 2.05 ms), i.e., with ±14.72° standard deviation of the angle.

### Phase-Dependent Modulation

**Figure 4:**
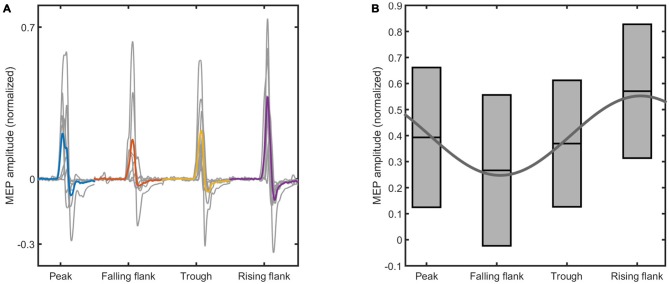
**The figure shows the raw motor evoked potential (MEP) data elicited at the end of the intervention. (A)** Shows the mean MEPs for each subject elicited at different phases of the tACS waveform (in gray), and the average across all the subjects (color coded). **(B)** Shows the boxplots obtained from the mean and standard deviation of the MEPs across all the subjects. The sinus is the result of the fitting of the mean MEP amplitude across the four tACS phases. The phase conditions and the normalized MEP amplitudes are indicated on the *x*-axis and the *y*-axis of both figures, respectively.

**Figure 5:**
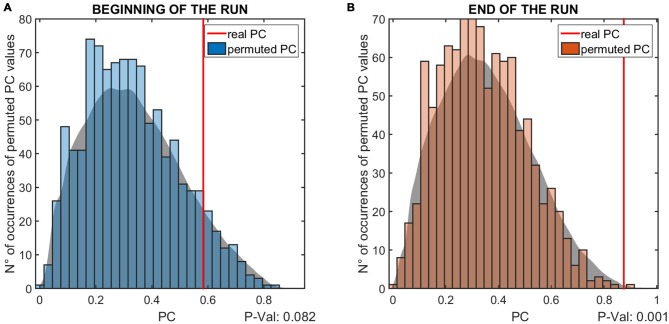
**The results of the permutation test for the phase coherence (PC) values of the MEP modulation is shown.** The two panels show the results relative to the first **(A)** and last **(B)** three elicited MEPs. The vertical red lines indicate the PC value resulting from the real data, while the histogram shows the distribution of values obtained with the permutation test. The gray patch is a smoothed version of the histogram, to better highlight the distribution of PC values. The *P*-values below the panels indicate the probability that the PC values obtained from the analysis are lower than the permuted values, i.e., are due to measurement noise.

The data shows a phase-dependent modulation of the MEPs at the end of the intervention (Figure 4). Statistical analysis (Figure 5) reveals no evidence of a phase-dependent modulation of the first MEPs (*p* = 0.082). The PC was well within the distribution of the values obtained with the permutation. In contrast, the PC of the last three MEPs showed a significant and strong phase alignment across the seven subjects (*p* = 0.001). Please note that the individuals’ phase lag in the final three trials was always negative and did not differ significantly from −90° (CI95*%* = −40.37° to −99.61°).

## Discussion

### Phase and Temporal Precision

In the present work, we describe a method for investigating the phase-dependency of TMS. Phase-dependent approaches require considerably higher temporal precision than closed-loop TMS on the basis of cortical band-power (Takemi et al., 2013; Gharabaghi et al., 2014; Kraus et al., 2016b). A number of approaches has been employed, most of which are based on *post hoc* assessment of the oscillatory phase (van Elswijk et al., 2010; Ferreri et al., 2011; Keil et al., 2013; Schulz et al., 2014; Berger et al., 2014; Kundu et al., 2014). A smaller number of studies employed closed-loop stimulation, by online triggering of the stimulation at the desired phase of the EEG (Bergmann et al., 2012; Zrenner et al., 2015b) or by combining tACS with TMS to control the phase at which stimulation should take place (Bergmann et al., 2009; Goldsworthy et al., 2016). In earlier approaches using tACS-TMS, the exact method for achieving phase-precise stimulation remains ambiguous. Moreover, reports of the precision achieved are rare. One study reports 1 ms jitter by using dedicated real-time hardware (Zrenner et al., 2015a), which is comparable with the 2 ms precision achieved by applying regular clinical hardware in our approach.

Perfect temporal precision can obviously only be achieved if all components run in a fully deterministic environment. However, this is often not the case, and labs do not have full control or knowledge about the precision of stimulation and recording devices. Without calibrations, the actual timing of the full system is affected by the behavior of the non-deterministic components, which can, at worst, cause a systematic bias. Furthermore, if medical certification of the devices is necessary, the desired control over certified components or the purchasing of dedicated and costly real-time recording hardware might not be feasible. The control approach presented here addresses precision, predictability and speed of the closed-loop system in three ways: first, by calibrating the set-up with a test pulse, second, by shifting the stimulation in time when the phase-delay is too large and third, by validating the system using a synchronous measurement of the tACS signal and the TMS-pulse artifact. The whole system can be easily implemented even if different hardware components are employed. The calibration is deemed to be particularly advantageous, since it allows for variability in communication delay, e.g., when different recording PCs, TCS or TMS hardware are being used. Additionally, by shifting the stimulation by a fixed phase-lag (2*π) the pulse can be triggered in an even more flexible real-time environment, e.g., when the desired phase cannot be hit because of the intrinsic delay of the system. Finally, the synchronous recording enables us to check individual trials and weigh or discard them according to the achieved precision.

### Phase-Dependent Modulation

Notably, when applied with 20 Hz tACS, the approach led to physiologically plausible results with regard to corticospinal excitability. Studies based on random stimulation found significant differences in the pre-stimulus beta-phase between high and low MEPs in occipital, but not in sensorimotor regions (Mäki and Ilmoniemi, 2010). Other studies reported significant angular-linear correlation between phase and MEP amplitude over the sensorimotor region only (Keil et al., 2013). The phase of beta oscillations has been shown to be decisive for cortical and corticospinal computations and has also been linked with excitability of the corticospinal system (Miller et al., 2012; Aumann and Prut, 2015; Romei et al., 2016). Furthermore, 20 Hz tACS affects movement acceleration (Pogosyan et al., 2009), and unlike other frequencies, increases corticospinal excitability at rest (Feurra et al., 2013).

The physiological analysis in this study was exploratory and preliminary. However, the results suggest that phase-modulation occurs with the cumulative duration of the tACS. More specifically, we found no evidence for modulation during the first few TMS pulses, but a significant modulation during the last few pulses, with a distinct phase shift of approximately −90°. Please note that the current through a capacitor leads the voltage by 90° (Horowitz and Hill, 1989), which therefore suggest that the instantaneous current, and not the voltage, drives the cortical excitability during tACS.

Of course, the exploratory sample size used in this methodological feasibility study and the lack of direct cortical recordings do not permit us to draw too many far-reaching conclusions from these results. Nevertheless, the present findings validate the feasibility of the proposed approach, demonstrating that it is possible to apply phase-dependent stimulation with high precision.

### Outlook

It is conceivable that the dot-product for the Fourier transformation could be calculated by taking the actual phases rather than the evenly spaced target phases. Depending on the noise level and its exact distribution in the estimation, this could reduce or increase the precision of the subsequent estimation of phase consistency and lag accordingly. Considering that the system has already achieved a good precision with regard to the targeted phases, we currently suggest that standard approaches to Fourier transformation be employed.

We are currently conducting a larger study, in which the interaction between phase and intensity of the TMS is being investigated. Many alternative research questions may be explored with this approach. For example, different phase lags could be explored for different frequencies to gain a better understanding of the response of the transcranial passage; or to ascertain whether there is a phase-alignment or a phase-drift over time thereby suggesting interactions with intrinsic frequencies.

## Conclusion

We presented a combination of tACS and TMS that achieved high temporal and phase precision even when implemented with regular and (partially) non-deterministic hardware. We found preliminary evidence for phase-dependent effects of TMS leading at roughly 90° and therefore suggesting that effects are current driven rather than voltage driven. Future studies might explore these properties with regard to their entrainment, accumulation and interaction with stimulation intensity.

## Author Contributions

VR designed and performed research, analyzed data and wrote the article. RB analyzed data and wrote the article. ST performed research and edited the article. AG designed research and wrote the article.

## Conflict of Interest Statement

The authors declare that the research was conducted in the absence of any commercial or financial relationships that could be construed as a potential conflict of interest.
